# Spectrophotometric Determination of *p*-Nitrophenol under ENP Interference

**DOI:** 10.1155/2021/6682722

**Published:** 2021-01-07

**Authors:** Hui Xia, Wenjing Zhang, Zhijie Yang, Zhenxue Dai, Yuesuo Yang

**Affiliations:** ^1^Key Lab of Eco-Restoration of Regional Contaminated Environment (Shenyang University), Ministry of Education, Shenyang 110044, China; ^2^Green Catalysis Center and College of Chemistry, Zhengzhou University, Zhengzhou 450001, China; ^3^Key Lab of Groundwater and Environment (Jilin University), Ministry of Education, Changchun 130021, China

## Abstract

Engineered nanoparticles (ENPs) have been widely developed in various fields in recent years, resulting in an increasing occurrence of nanoparticles in the natural environment. However, the tiny substances have created unexpected confusion in environmental sample testing due to the negative nanoeffect of ENPs. In this paper, a novel technique of spectrophotometric determination of *p*-nitrophenol (PNP) was developed under the interfering impact of nano-Fe(OH)_3_, widely distributed in the natural environment as a typical example of ENPs. Because of the strong absorption at the two characteristic peaks of PNP, namely, 317 nm and 400 nm, nano-Fe(OH)_3_ interfered with the colorimetric determination of PNP. Thus, the developed testing method, with HCl acidification at 60°C and ascorbic acid (AA) masking FeCl_3_, was proposed and successfully realized the accurate determination of PNP in water samples by ultraviolet spectrophotometry with 317 nm as the absorption wavelength. The final colorimetric system of 5% HCl, 10% CH_3_OH, and 1% ascorbic acid was confirmed by optimized batch experiments, and the optimum condition of acidification pretreatment was heating at 60°C for 20 min. Further results demonstrated that the proposed novel method had good accuracy and reproducibility even in high-salinity natural water bodies such as groundwater and surface water. The testing technique presented in this paper provided an interesting and useful tool for problem solving of PNP surveys under ENPs' interference and practically supported water quality assessment for a better environment.

## 1. Introduction

*p*-nitrophenol (PNP), a class of highly toxic and environmental persistent organic pollutants (POPs), is not easily biodegradable or naturally photolyzed; it accumulates and causes long-term damage to the environment, so PNP is one of the most frequently detected organic pollutants [1, 2]. In view of the environmental toxicity and chemical inactivity, PNP has been often used as a typical representative of POPs, and more and more environmental workers have begun to carry out environmental engineering treatment and laboratory research work on PNP. However, before carrying out the related work, the first key issue was how to find a simple, economical, and accurate test method. At present, the relatively mature test methods for PNP included UV-vis spectrophotometry [3, 4], high-performance liquid chromatography [5, 6], gas chromatography-mass spectrometry [7, 8], and electrochemical assessment [9–11]. Among them, UV-vis spectrophotometry has the advantages of simple operation, rapidity, low labor intensity, and high analysis efficiency. More importantly, the instrument used in the method was cheap, and almost all chemical laboratories were equipped to use it. However, it should be noted that there are more and more engineered nanoparticles (ENPs) in environmental water samples due to the rise of nanotechnology, and the nanosubstances bring unexpected trouble to the traditional analysis and testing of POPs. For example, iron-based ENPs will interfere with the UV-vis spectrophotometric determination of PNP.

**Figure 1:**
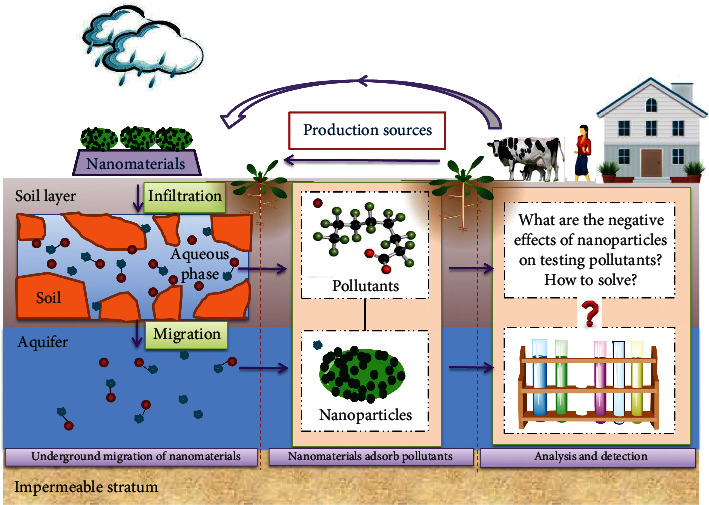
Significance of testing in the comigration of ENPs and environmental pollutants.

Actually, nanotechnology has revolutionized various research fields, andENPs have found broader application in environmental remediation [12–14], new energy [15], biomedicine [16], daily consumer goods [17, 18], agriculture [19], etc., due to their extensive potentiality and versatility in the past decade [20]. As shown in Figure 1, our research group [21–23] found that these emerging ENPs enter into the groundwater and surface water bodies by atmospheric sedimentation, surface runoff, underground infiltration, etc. However, the ENPs would be adsorbed in environmental media or comigrate with environmental pollutants due to their unique nanoproperties, such as huge specific surface area, strong adsorption, catalysis, chelating ability, etc. [24, 25]. As a result, a new type of stable nanocolloid was formed by the ENPs in groundwater and surface water systems and stayed in the environment for a long time. Unfortunately, the nanocolloid would change the light paths, which might limit the application of analytical methods based on spectral theory. So, it was indispensable and of great significance to study the ENPs' influence on the accuracy and stability of the analysis results when carrying out pollutant testing work in the groundwater and surface water bodies.

For the past couple of years, environmental remediation technology based on metallic iron ENPs has been investigated as a new tool for water and soil treatment and has gradually been accepted and commercialized in many countries due to its effectiveness in the removal of pollutants as well as the low cost of production [26, 27]. For example, the pollution remediation agent nano-Fe^0^ has the advantages of high efficiency, low cost, low toxicity, etc., and has received extensive attention in the in situ remediation of heavy metal and organic pollutants in soil [28]. Nano-Fe_3_O_4_, as a heterogeneous catalyst, was evaluated to activate Na_2_S_2_O_8_, which was proven to be an efficient and promising agent for the treatment of leachate biochemical effluent [14]. Bagbi and coworkers [29] successfully synthesized L-cysteine-functionalized Fe_3_O_4_ nanoparticles that could be reused for lead and chromium adsorption removal. He and his coworkers [30] reported nanoscale zero-valency iron/nickel supported on zeolite for the simultaneous removal of nitrate and phosphate from an aqueous solution. Chen and coworkers [31] concluded that nano-FeS could not only remove redox-sensitive pollutants by a chemical redox reaction, but could also treat pollutants by chemical adsorption due to its larger specific surface area and higher reactivity than macro-FeS.

In the process of water treatment, Fe(OH)_3_ nanoparticles gradually evolve from the above iron-based ENPs and remain in the aqueous solution for a long time in the form of brown-red Fe(OH)_3_ colloids. The Fe(OH)_3_ colloid has strong absorption at the characteristic absorption peaks of PNP (317 nm and 400 nm), which interferes with the test results of UV-Vis spectrophotometry. In this paper, an improved technique for the spectrophotometric determination of PNP, as a typical POPs, is developed under interfering conditions of nano-Fe(OH)_3_, as a typical example of ENPs in a natural aqueous environment. HCl acidification at 60°C and ascorbic acid (AA) masking of FeCl₃ were conducted in order to eliminate the negative effect of Fe(OH)_3_ on the spectrophotometric determination of PNP. The influencing factors such as heating time, HCl dosage, temperature, and common anions and cations were optimized through batch experiments. Finally, the accuracy of the improved testing method was verified by standard experiments, and the precision was investigated in several repeated experiments. This work provided robust results to support the environmental scientists' further investigations into both the treatment of POPs and the cotransport of ENPs and organic contaminants.

## 2. Materials and Methods

### 2.1. Chemicals and Reagents

*p*-nitrophenol (C_6_H_5_NO_3_, Cas: 100-02-7, Macklin Biochemical Technology Co., Ltd., Shanghai, China, the physical and chemical properties are shown in Table 1); hydrochloric acid (HCl, Beijing Chemical Plant Co., Ltd., Beijing, China); methanol (CH_3_OH, high-performance liquid chromatography grade, Thermo Fisher Scientific, Waltham, USA); sodium bicarbonate (NaHCO_3_, Sigma Aldrich (Shanghai) Trading Co., Ltd., Shanghai, China); ascorbic acid (C_6_H_8_O_6_, Sinopharm Chemical Reagent Co., Ltd., Shanghai, China); calcium chloride (CaCl_2_), manganese sulfate (MnSO_4_·H_2_O), potassium nitrate (KNO_3_), ferric chloride (FeCl_3_·6H_2_O), magnesium sulfate (MgSO_4_·7H_2_O), and potassium chloride (KCl) were purchased from Tianjin Bodi Chemical Co., Ltd., Tianjin, China. All the reagents involved in this article were of analytical grade or higher. All solutions were prepared with ultrapure water from a pure water treatment process (≥18 MΩ∙cm, Milli-Q Advantage A10, Millibo (Shanghai) Trading Co., Ltd., Shanghai, China).

### 2.2. Preparation of Experimental Solutions

**Figure 2:**
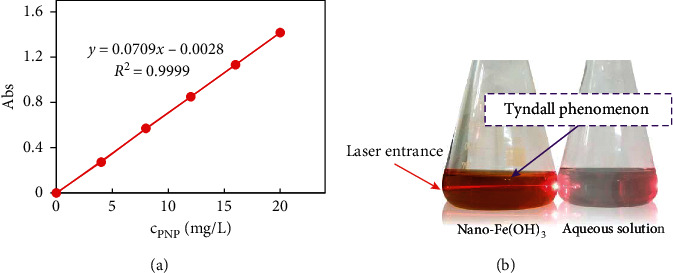
(a) PNP standard curve. (b) Tyndall phenomenon of the nano-Fe(OH)_3_ solution.

Stock solutions of PNP (1.000 g/L), NaHCO_3_ (3.440 g/L), MgSO_4_ (5.000 g/L), CaCl_2_ (2.774 g/L), KCl (0.956 g/L), MnSO_4_ (1.388 g/L), and KNO_3_ (1.628 g/L) were prepared in ultrapure water in order to match the salt content in natural water. The corresponding working solutions were obtained by diluting stock solutions daily in ultrapure water. The PNP standard solutions of 0.00 mg/L, 4.00 mg/L, 8.00 mg/L, 12.0 mg/L, 16.0 mg/L, and 20.0 mg/L were diluted step by step with the PNP stock solution. It should be noted that the six PNP standard solutions all contained 10% CH₃OH, 5% HCl, and 1 g/L ascorbic acid. Afterwards, the absorbance of PNP standard solutions was determined at 317 nm, which was the unique characteristic UV wavelength of PNP. The PNP standard curve was drawn with the concentration as the abscissa and absorbance as the ordinate, and the linear regression equation was obtained by fitting, as shown in Figure 2(a). It was concluded that the PNP standard curve was linear in the range of 0.00‒20.0 mg/L, and its correlation coefficient, *R*^*2*^, was above 0.999.

The nano-Fe(OH)₃ solution was prepared by extended hydrolysis of Fe^3+^. In detail, 0.0776 g FeCl_3_·6H_2_O was dissolved in 250 mL ultrapure water and hydrolyzed naturally at room temperature for more than four days. Before using the solution, the particle size and ζ potential of the nano-Fe(OH)_3_ solution were measured by a Particle Sizer and Zeta Potential Analyzer (Nano-ZS, Malvern, UK), as shown in Table 2. The average particle size of Fe(OH)_3_ was 39.2 nm, which proved that the solution was a nanocolloid. The average ζ potential was 35.3 mV, which implied that the nano-Fe(OH)_3_ system was very stable after extended hydrolysis [33, 34]. In order to further verify the existence of the colloid, a simple Tyndall experiment was carried out, as shown in Figure 2(b). A bright red “light channel,” which was the Tyndall phenomenon, was obviously observed when a laser was irradiated from the side of the solution. Therefore, it was proven that the nano-Fe(OH)₃, obtained by four-day natural hydrolysis, was suitable for the follow-up study of nanoparticles interfering with PNP tests.

### 2.3. Improved Analytical Methods for Assessing PNP in the Presence of Nano-Fe(OH)_3_

It was found that the existence of engineered nanoparticles, such as nano-Fe(OH)_3_, could lead to inaccurate spectrophotometric results because they formed stable nanomicelles in solution. It is universally known that the surface structure of colloidal particles could be broken, and their stability could be destroyed by adding electrolytes or heating. In this context, 5% HCl was added to the colorimetric system and heated at 60°C for 20 min in order to dispel the negative effect of nano-Fe(OH)_3_. The preparation and purification procedures were as follows.

**Figure 3:**
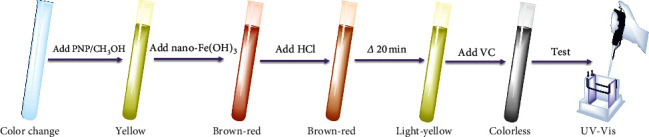
Experimental procedures and phenomena.

First, 1−5 mL of 40 mg/L PNP standard solution was sampled and injected into a 10 mL colorimetric tube, and 1.00 mL CH_3_OH, 2.00 mL nano-Fe(OH)_3_ solution, and 1.00 mL HCl (50%, v/v) were added in turn. An additional 2 mL of the corresponding ions' stock solutions was added for the experiment on coexisting ion interference. Afterwards, the samples were slightly warmed at 60 °C for 20 min, and then 1 mL of ascorbic acid solution (5 g/L) was added after cooling. Finally, the PNP was quantitatively analyzed at a wavelength of 317 nm, which was the characteristic UV peak under acidic conditions, as revealed by UV-visible spectrophotometry (Hitachi U-2910, Hitachi Ltd., Tokyo, Japan). The abovementioned procedures and experimental phenomena are shown in Figure 3.

## 3. Results and Discussion

### 3.1. Selection of Analytical Spectral Lines

**Figure 4:**
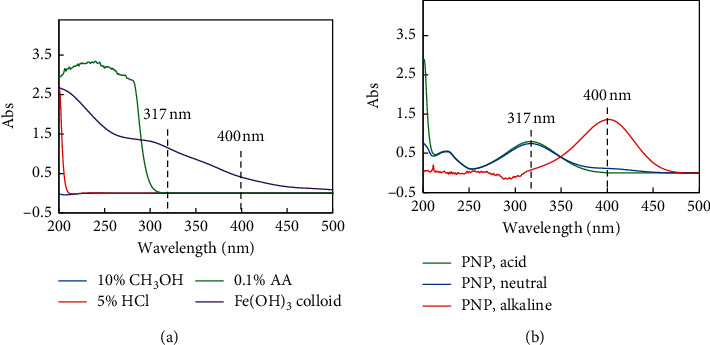
UV-Vis absorption spectra of (a) different reagents and (b) 20 mg/L PNP under different pH.

It can be seen from Figure 4(b) that *p*-nitrophenol had only one UV absorption peak of 317 nm under strongly acidic conditions (see the green line) and the solution was colorless, while there was only one absorption peak at 400 nm under strongly alkaline conditions (see the red line), and the solution was yellow. The two characteristic peaks coexist in a pure aqueous solution (see the blue line), and the color of the solution becomes lighter. At the same concentration, the absorbance of 400 nm in strong alkalinity was higher than that at 317 nm under strong acidity, which indicated that the sensitivity of colorimetry using 400 nm as a characteristic wavelength was higher. However, there were many heavy metals, such as Ca, Mg, Fe, etc., in natural water [35, 36]. Actually, these heavy metals would produce microscopic particles in an alkaline solution, which interfered with the accuracy of the determination. For example, the Fe ion, even at low concentrations, formed a colloid under alkaline conditions, which interfered with the results of the colorimetric determination (see Figure 4(a)). Because these metals are soluble under acidic conditions, 317 nm, which was the characteristic peak in acidic water, was selected as the analytical wavelength of PNP colorimetry in the actual testing work in order to avoid the interference of the metals.

### 3.2. Role of Methanol and Ascorbic Acid

As a common catalyst in the treatment of environmental pollution, metal Fe often catalyzed the production of high-energy hydroxyl radicals, which was used for the rapid and nonselective degradation of many organic pollutants [37–39]. Methanol was a classical quenching agent of hydroxyl radicals [40, 41], which could immediately interrupt the possible follow-up reaction of OH radicals, so as to obtain the accurate PNP concentration at the sampling time. Therefore, the addition of methanol during the testing process was to avoid the possible decomposition of PNP by hydroxyl radicals, which improved the application scope of the method.

In addition, it should be noted that the reagents, such as CH_3_OH, HCl, and AA, had no UV absorption at 317 nm (see the blue, red, and green lines in Figure 4(a)), so it did not affect the results of the colorimetric determination of PNP.

**Figure 5:**
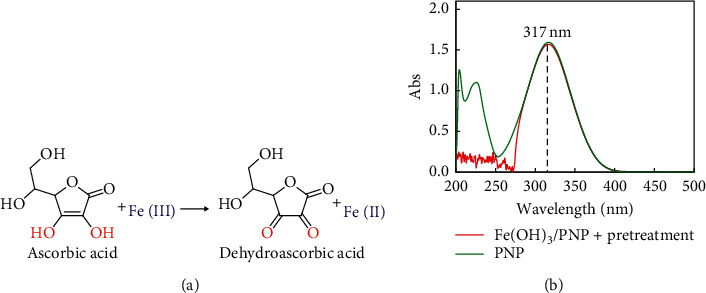
(a) Reduction of Fe(III) by AA. (b) Research on Fe(OH)_3_ interference elimination.

A yellowish FeCl_3_ was regenerated from nano-Fe(OH)_3_ after acidizing with hydrochloric acid and heating pretreatment. Unfortunately, the FeCl_3_ would enhance the UV absorption at 317 nm and still interfered with the colorimetric determination of PNP. After consulting the literature, it was found that ascorbic acid was a common masking reagent for Fe(III) [42]. The valence state of Fe ion could be changed, i.e., Fe(III) could be reduced to Fe(II), due to the reducibility of ascorbic acid, as shown in Figure 5(a). As can be seen from Figure 5(b), after blank zero adjustment, the scanning peak shape between 280 nm and 500 nm of PNP with Fe(OH)_3_ after AA reduction treatment was basically the same as that of PNP under acidic conditions, and the absorbance at 317 nm was completely the same, which shows that the addition of ascorbic acid completely eliminated the FeCl_3_ interference. As a result, there was a prerequisite for the quantitative analysis of PNP.

### 3.3. Effect of Nano-Fe(OH)_3_ and Coexisting Ions

In the groundwater and surface water, K⁺, Na⁺, Ca^2^⁺, Mg^2^⁺, Fe³⁺, Mn^2^⁺, HCO_3_^−^, SO_4_^2−^,NO_3_^−^, and Cl⁻ were the most common ions [43, 44]. In order to study the interference of these ions with the improved method, the proportions were set according to Table 3, and standard addition batch experiments were carried out. According to the results in Table 3, when there was K⁺, Na⁺, Ca^2^⁺, Mg^2^⁺, Fe³⁺, Mn^2^⁺, HCO_3_^−^, SO_4_^2−^, NO_3_^−^, and Cl⁻ in the colorimetric system, the recoveries of all the experiments were in the range of 101‒105%, in line with our expectations indicates that the existence of these chemicals did not interfere with the results of PNP colorimetric determination. Moreover, when nano-Fe(OH)_3_ was added to the system, the recovery of PNP was 103.9%, which indicated that the interference had been eliminated by the improved method.

### 3.4. Optimization of HCl and AA Dosage

**Figure 6:**
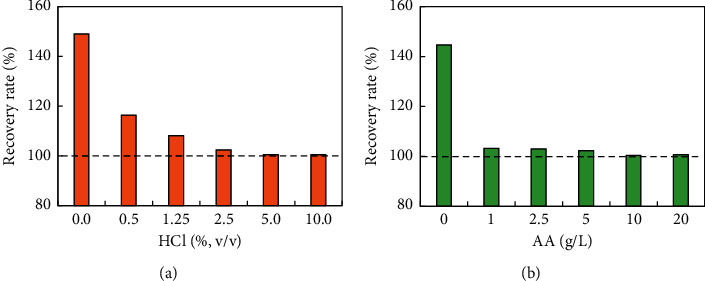
(a) Optimization of HCl dosage. (b) Optimization of AA dosage.

There were two functions of HCl in the colorimetric system. On the one hand, the solution was acidic due to the addition of HCl, so the ultraviolet absorption of PNP reached a maximum at 317 nm, which allowed for effective quantitative colorimetry. On the other hand, the stability of nano-Fe(OH)_3_ was destroyed by HCl due to their neutralization reaction, thus achieving the purpose of eliminating nano-Fe(OH)_3_ interference. As shown in Figure 6(a), because the interference of Fe is not deducted, the recovery rate of PNP was 149% without adding HCl, which indicated that the PNP test result was incorrect, whereas the recovery rates were 116%, 108%, 102%, 101%, and 101%, respectively, at HCl = 0.5%, 1.25%, 2.5%, 5%, and 10%. It was clear that the recovery rate was close to 100% when the amount of HCl was more than 5%. Therefore, the reasonable use of HCl should be greater than 5% in the determination process.

As can be seen from Figure 6(b), the recovery rates were 145%, 103%, 103%, 102%, 100%, and 101% at AA = 0 g/L, 1 g/L, 2.5 g/L, 5 g/L, 10 g/L, and 20 g/L, respectively, which indicated that the usage of AA should be controlled at about 10 g/L.

### 3.5. Heating Temperature and Time

**Figure 7:**
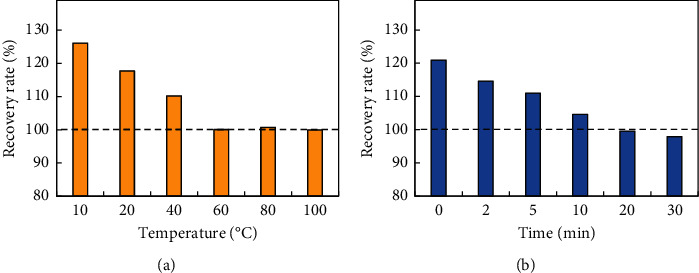
(a) Optimization of heating temperature. (b) Optimization of heating time.

Heating temperature was an important factor affecting the accuracy of the PNP determination. In Figure 7(a), all the recovery rates of PNP were more than 110% at *T* < 60°C, while the recovery rates were close to 100% at *T* ≥ 60°C. However, too high a temperature might affect the chemical properties of the organic pollutant, which is not conducive to the determination of the pollutant. Synthetically, 60°C was confirmed as the ideal heating temperature in the improved method. Similarly, the heating time of 20 min is best according to the results shown in Figure 7(b).

### 3.6. Accuracy and Precision

As we all know, the concentration of PNP varies over a wide range in no matter groundwater or surface water. In order to simulate the complicated PNP contaminated samples as approximately as possible, a batch of PNP samples containing nano-Fe(OH)_3_ with different known concentrations were prepared and tested. The accuracy and precision were determined according to the method proposed in this paper. Each sample was repeated 12 times to study the accuracy and precision of the proposed method, and the investigation results are shown in Table 4. Scientifically, the accuracy and relative standard deviation of the method were less than 3% and 6%, respectively, which could meet the requirements of practical testing.

### 3.7. Environmental Implication

In this paper, the problem of Fe(OH)_3_ nanoparticles interfering with the colorimetric test of PNP has been solved successfully, and the proposed method is simple, reliable, and applicable. It is demonstrated that the heating-acidizing method works well in destroying the stable nanocolloids, which results in the elimination of the interference of ENP and accuracy improvement of POP determination. Although it is not a so advanced technique, the UV-Vis spectrophotometer is available in almost all laboratories due its low price and practicability. Therefore, this research could provide new ideas for both environmental workers and laboratory staff who are not equipped with large-scale equipment, and it has more extensive application value.

The reported method had not been used to treat real samples, but got successful applications in our subsequent research of scientific samples. Actually, Fe-based nanomaterials had been demonstrated to be effective catalyst for remediation of groundwater/surface water which were contaminated by PNP or some other organic species. However, the determined concentration of PNP was always inaccurate by spectrophotometry because the interference of Fe(OH)_3_ nanoparticles which was gradually evolved from the Fe-based nanomaterials. This technical challenge had been perfectly solved by this improved method, and as a methodology study, this work provided a technical guarantee as well for the remediation from other nanoparticles.

Actually, because of the burgeoning nanotechnology, there may be not only Fe-based nanoparticles, but also Ag nanoparticles [45], Au nanoparticles [46], TiO_2_ nanoparticles [47], Cu-based nanoparticles [48], etc., in the various samples. It is possible that these engineered nanoparticles interfere with the accuracy of the pollutant testing. In this case, heating-acidification can be used to destroy the stability of the nanoparticles so that a better and more accurate determination can be achieved.

To sum up the above arguments, the most important advantage of the proposed method over other spectrophotometric methods is to realize the colorimetric determination of environmental pollutants under the interference of ENPs, which greatly expands application scope of spectrophotometry technology.

## 4. Conclusions

In this paper, methanol was used to quench high-energy free radicals, nano-Fe(OH)_3_ colloid was destroyed by HCl at medium temperature, and ascorbic acid was used to mask FeCl_3_. We successfully established a new, accurate colorimetric method for the determination of PNP in the presence of Fe(OH)_3_ nanoparticles. The results of batch experiments showed that the most suitable conditions for the colorimetric system were that the dosages of HCl, ascorbic acid, and methanol were 5%, 1 g/L, and 10%, respectively, the temperature of acidizing pretreatment was 60°C, and the heating time was 20 min. Moreover, it was proven that PNP could be tested simply and effectively, even in high-salinity water samples. Both the accuracy and the precision of the proposed method were good, and they could meet the requirements of actual samples. Scientifically, this work provided new ideas for analysis and testing in the presence of engineered nanoparticles.

## Figures and Tables

**Table 1:** The physical and chemical properties of *p*-nitrophenol [32].

Parameter	PNP	Parameter	PNP
Molecular formula	C_6_H_5_NO_3_	Molar mass	139.11 g/mol
pKa	7.15 (25°C)	Water solubility	1.6 g/L (25 °C)
Melting point	113‒114°C	Boiling point	279 °C
LD_50_	250 mg/kg (rats, orally)	Octanol/water partition coefficient	1.91

Molecular structure in acid solution (colorless)		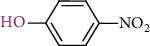	
Molecular structure in alkaline solution (yellow)		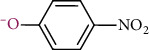	

**Table 2:** The properties of the nano-Fe(OH)_3_ solution.

Reagents	Times	Particle size (d.nm)	Average (d.nm)	Zeta potential (mV)	Average (mV)
Nano-Fe(OH)_3_	1	38.80	39.2	31.7	35.4
	2	38.41		37.4	
	3	40.54		37.2	

**Table 3:** The nano-Fe(OH)_3_ and coexisting ions interference test.

Reagents	Nano-Fe(OH)_3_	NaHCO_3_	MgSO_4_	CaCl_2_	KCl	MnSO_4_	KNO_3_
Cations addition (mg/L)	10^1^	188	200	200	100	100	126
Anions addition (mg/L)	3	500	800	355	182	177	200
PNP standard addition (mg/L)	12	12	12	12	12	12	12
Measured value of PNP standard addition (mg/L)	12.46	12.17	12.43	12.05	12.22	12.27	12.50
Standard recovery rate (%)	103.9	101.4	103.6	100.4	101.8	102.3	104.2

^1^The amount of nano-Fe(OH)_3_ added was calculated in terms of Fe.

**Table 4:** The results of accuracy and precision.

Serial no.	Preparation sample (mg/L)	Times	Mean (mg/L)	Accuracy (%)	SD^1^	RSD^2^ (%)
1	2	12	2.06	2.93	0.11	5.31
2	4	12	3.94	‒1.39	0.20	5.17
3	6	12	6.05	0.85	0.22	3.61
4	8	12	8.13	1.59	0.17	2.11
5	10	12	9.92	‒0.76	0.15	1.48
6	12	12	11.94	‒0.47	0.09	0.75
7	14	12	14.02	0.12	0.12	0.87
8	16	12	15.79	‒1.30	0.13	0.80
8	20	12	20.23	1.15	0.49	2.44
10	30	12	30.09	0.30	0.35	1.15
11	40	12	40.14	0.34	0.42	1.06
12	50	12	50.70	1.40	0.48	0.94

^1^SD: standard deviation; ^2^RSD: relative standard deviation.

## Data Availability

The data used to support the results of this study are included within the article. Any further information is available from the corresponding author upon request.
